# Diagnosis and Management of Middle Ear Neuroendocrine Tumour (MeNET)

**DOI:** 10.3390/life16081286

**Published:** 2026-08-04

**Authors:** Magdalena Chomczyńska, Andrzej Kucharski, Anna Szymańska, Agnieszka Korolczuk, Marcin Szymański

**Affiliations:** 1 Department of Otolaryngology Head and Neck Surgery, Medical University of Lublin, 20-954 Lublin, Polandmarcin.szymanski@umlub.pl (M.S.); 2 Department of Interventional Radiology and Neuroradiology, Medical University of Lublin, 20-954 Lublin, Poland; 3 Department of Clinical Pathomorphology, Medical University of Lublin, 20-954 Lublin, Poland

**Keywords:** MeNET, middle ear, neuroendocrine tumour

## Abstract

Middle ear neuroendocrine tumours (MeNETs) are rare epithelial neoplasms with neuroendocrine differentiation that pose significant diagnostic and therapeutic challenges. The clinical presentation of MeNETs is often nonspecific and can mimic other middle ear pathologies, such as chronic otitis media, cholesteatoma, or paraganglioma Common symptoms include conductive hearing loss, otalgia, intermittent or persistent tinnitus, ear fullness, and dizziness. We present five patients who underwent surgery in our University Otolaryngology Centre between 2019 and 2025, in whom histopathological examination confirmed the diagnosis of MeNET. Although MeNET is typically considered an indolent tumour, rare cases of locally aggressive behaviour and distant metastases have been reported in the literature. Metastatic potential appears to correlate with histopathological features such as increased mitotic activity, Ki-67 proliferation index > 5%. The treatment of choice for MeNET is surgical resection of the tumour, with the choice of surgical technique depending on the stage of the tumour, its relationship to surrounding anatomical structures, and the possibility of hearing preservation. In our study, we highlighted the importance of radical tumour excision to minimize the risk of recurrence. Given the risk of recurrence and the risk of potential metastases, long-term follow-up is necessary, particularly in patients with advanced-stage tumours.

## 1. Introduction

Middle ear neuroendocrine tumour (MeNET) is a rare and diagnostically challenging entity. Despite increasing recognition of MeNETs, their histogenesis remains controversial. Historically, these tumours have been referred to as middle ear adenoma, carcinoid tumour of the middle ear, adenomatous tumour of the middle ear, and neuroendocrine adenoma of the middle ear. According to the WHO Classification of Head and Neck Tumours (5th edition, 2022), these lesions are recognized as epithelial neoplasms with neuroendocrine differentiation and have historically been referred to by various names [1]. In the present study, we consistently use the term MeNET, which is increasingly adopted in recent literature [2]. The use of a unified terminology is important to reduce the longstanding inconsistency in nomenclature and to facilitate comparison between published studies. More recently, Stojanov and Asa emphasized the concept of a unified neuroendocrine entity and proposed the term Mixed Adenoma and Well-Differentiated Neuroendocrine Tumour (MANET) of the Middle Ear, further supporting the view that these lesions represent a single clinicopathological entity with a spectrum of histological features [2]. MeNETs account for less than 2% of all middle ear neoplasms [3].

Despite their low malignant potential, MeNETs can cause significant morbidity due to their location and potential for local invasion. The clinical presentation of MeNETs is often nonspecific and can mimic other middle ear pathologies, such as chronic otitis media, cholesteatoma, or paraganglioma. Common symptoms include conductive hearing loss, otalgia, intermittent or persistent tinnitus, ear fullness, and dizziness. In advanced cases, aural discharge and facial nerve paralysis may occur [4]. MeNETs present a diagnostic challenge due to their overlapping clinical and radiological features. MeNET must be differentiated from cholesteatoma, glomus tympanicum, jugulotympanic paraganglioma, acoustic neuroma, meningioma, endolymphatic sac papillary tumour, and adenocarcinoma [5,6,7].

The rarity of this tumour significantly limits the availability of standardized diagnostic and therapeutic guidelines. Recent reviews additionally emphasized the ongoing controversy regarding nomenclature, histogenesis, and optimal management strategies in MeNETs [8]. Most available evidence is based on isolated case reports and small retrospective series [9]. Imaging findings are often nonspecific and may resemble other middle ear lesions.

Radiological assessment plays a central role in the diagnostic workup of MeNET. High-resolution computed tomography (HRCT) is useful for evaluating tumour extension, ossicular chain involvement, mastoid infiltration, and possible bone erosion. Unlike cholesteatoma, MeNET usually demonstrates preservation of surrounding bony structures despite occupying large portions of the tympanic cavity and mastoid air cell system [9,10]. MRI complements CT by providing superior soft tissue characterization. Typical MRI findings include hypointense or isointense signal on T2-weighted sequences and moderate to intense contrast enhancement following gadolinium administration. However, these features are not pathognomonic and overlap with paragangliomas and inflammatory lesions [5,6,9].

Histopathological examination with immunohistochemical profiling remains essential for definitive diagnosis. The most consistently expressed neuroendocrine markers include synaptophysin and chromogranin A, while cytokeratin positivity confirms epithelial differentiation. Additional markers, including CD56, neuron-specific enolase (NSE), serotonin, pancreatic polypeptide, and the Ki-67 proliferation index may provide supplementary diagnostic and prognostic information. In most reported cases, the Ki-67 index remains low, supporting the indolent nature of these tumours. Nevertheless, increased proliferative activity may correlate with more aggressive behaviour and metastatic potential.

From a surgical perspective, complete excision remains the cornerstone of treatment. The extent of surgery depends on tumour stage, hearing status, ossicular involvement, extension into the mastoid cavity, and proximity to critical neurovascular structures. Both transcanal and mastoid approaches have been described, including canal wall-up and canal wall-down procedures. Preservation of hearing structures should be attempted whenever oncologically feasible. However, complete tumour removal may necessitate ossicular chain disassembly or tympanic membrane resection in advanced lesions.

A systematic review published in recent years highlighted the lack of standardized management protocols and emphasized the importance of complete surgical excision and long-term follow-up in MeNET patients [9].

## 2. Materials and Methods

We conducted a retrospective analysis of five consecutive patients with histopathologically confirmed MeNET who underwent surgical treatment at the Department of Otolaryngology and Pediatric Otolaryngology, Medical University of Lublin, between January 2019 and January 2025. The study group consisted of three women and two men aged 19–49 years. Detailed demographic, clinical, radiological, pathological, treatment, and follow-up data are presented in Table 1.

Inclusion criteria comprised histopathological confirmation of MeNET and availability of complete clinical, radiological, surgical, and follow-up data. No patients meeting these criteria were excluded. Demographic characteristics, presenting symptoms, otoscopic findings, audiological assessment, radiological studies, surgical reports, histopathological findings, immunohistochemical results, and follow-up data were retrospectively reviewed using electronic medical records.

Audiological evaluation included pure-tone audiometry and tympanometry. Radiological assessment consisted of high-resolution computed tomography (HRCT) in all patients, with magnetic resonance imaging (MRI) performed when required for preoperative assessment. Tumour stage was determined according to the Marinelli staging system.

Follow-up consisted of regular outpatient otolaryngological examinations, audiological assessment, and radiological imaging. According to our institutional follow-up protocol, temporal bone CT is routinely scheduled approximately 6 months after surgery, followed by annual MRI examinations. The duration of follow-up ranged from 8 months to 6.5 years. Owing to the rarity of MeNET, additional surveillance was individualized according to the patient’s clinical course and imaging findings when clinically indicated.

In addition, a narrative review of the English-language literature concerning middle ear neuroendocrine tumours was conducted. A search of the PubMed, Scopus, and Web of Science databases was performed for studies published between 1984 and 2025 using the following keywords: “middle ear adenoma”, “middle ear neuroendocrine tumour”, “middle ear adenomatous neuroendocrine tumour”, “MeNET”, and “neuroendocrine adenoma of the middle ear”. Case reports, case series, review articles, and studies describing clinical presentation, radiological findings, histopathological characteristics, treatment strategies, and long-term outcomes were included. Non-English publications, conference abstracts without full text, and duplicate reports were excluded. The retrieved literature was reviewed narratively to summarize current knowledge regarding the diagnosis, management, and prognosis of MeNET.

## 3. Results

### 3.1. Case 1

The patient (A.P.), a 38-year-old female, was admitted to our department due to a tumour in the left tympanic cavity with suspected paraganglioma. The patient reported a left-sided ear fullness and conductive hearing loss. Otoscopic examination revealed a dark red tumour behind the tympanic membrane of the left ear.

**Figure 1:**
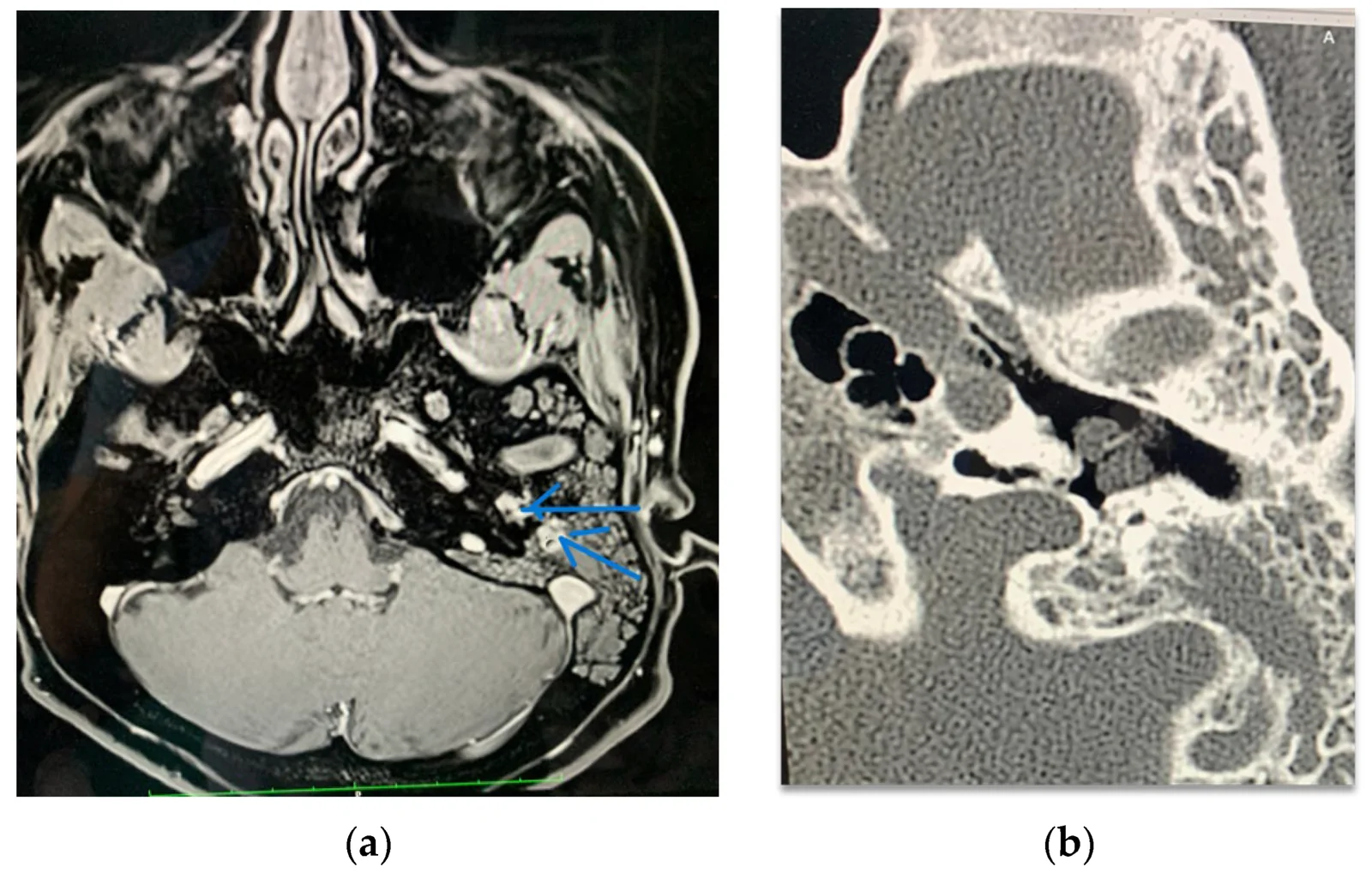
Imaging studies performed on Patient 1 (A.P.): (**a**) MRI scan—the arrows show a focal lesion in the left middle ear; (**b**) The CT scan of the left ear.

MRI showed an irregular focal lesion within the left middle ear at the level of the promontory, filling the middle ear cavity, exhibiting reduced signal intensity (SI) on T2-weighted (T2W) sequences and increased SI on T1-weighted (T1W) sequences (Figure 1a). The lesion demonstrated intense, heterogeneous enhancement following intravenous contrast administration. The lesion dimensions were 15 × 11 × 9 mm. Temporal bone CT imaging performed on the patient revealed a focal lesion in the left middle ear accompanied by exudative-inflammatory changes in the left middle ear and mastoid air cells. No evidence of bone destruction involving the scutum or significant destruction of the auditory ossicles was observed. No clear destruction of the bony labyrinth was identified (Figure 1b).

**Figure 2:**
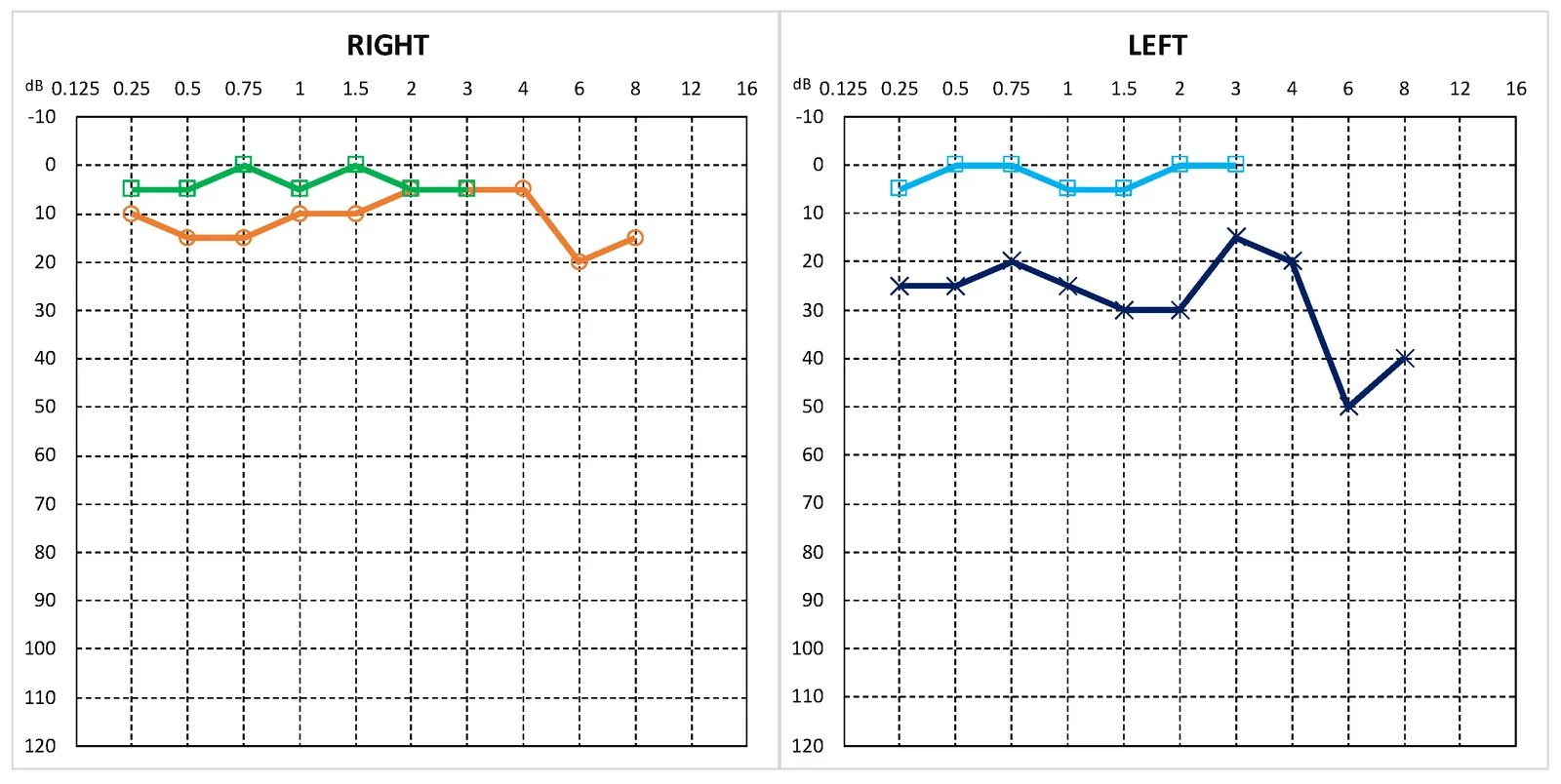
The result of pure-tone audiometry of the patient revealed conductive hearing loss in the left ear. Green and orange lines represent air-conduction and bone-conduction thresholds of the right ear, respectively. Dark blue and light blue lines represent air-conduction and bone-conduction thresholds of the left ear, respectively.

**Figure 3:**
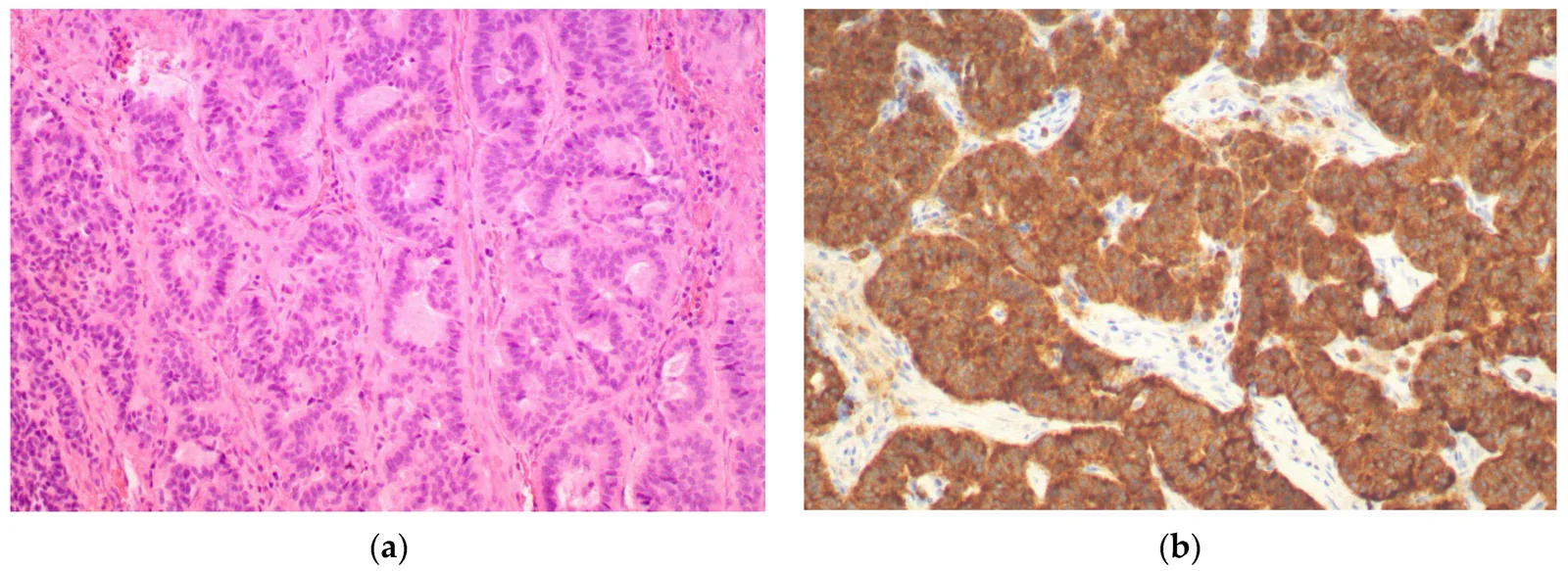
Middle ear neuroendocrine tumour G1: (**a**) H + E ×400; (**b**) Immunohistochemical expression of synaptophysin. ×400.

The patient presented with conductive hearing loss with ABG (air–bone gap) ranging between 15–25 dB (Figure 2). A canal wall-up mastoidectomy was performed in a large mastoid process filled with mucus. A multinodular tumour was identified, filling the antrum and partially occupying the mastoid process. The tumour extended into the attic and occupied the majority of the tympanic cavity, surrounding the ossicles. The tumour extended with projections into numerous mastoid cells without bone infiltration. The Marinelli stage of the tumour was T2b. The histopathological examination showed middle ear neuroendocrine tumour G1 (MeNET G1). Immunohistochemistry demonstrated: chromogranin+, synaptophysin+, CD56+, and the Ki-67 index was about 2% (Figure 3).

### 3.2. Case 2

A 49-year-old male patient (P.M.) presented with a right temporal bone tumour. He reported hearing loss in the right ear for several years, with no history of ear discharge or vertigo.

**Figure 4:**
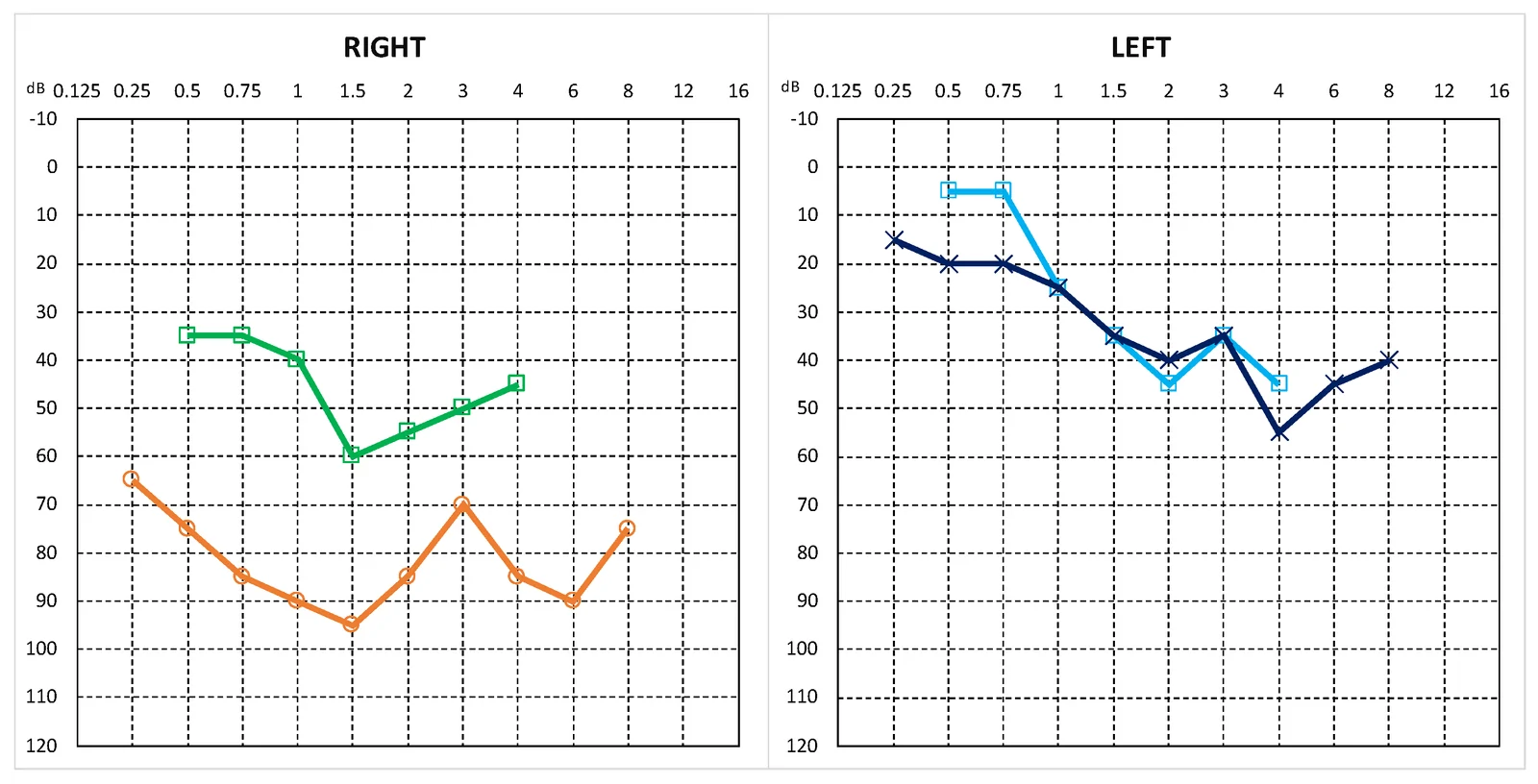
The results of pure-tone audiometry indicated mixed hearing loss in the right ear. Green and orange lines represent air-conduction and bone-conduction thresholds of the right ear, respectively. Dark blue and light blue lines represent air-conduction and bone-conduction thresholds of the left ear, respectively.

Otoscopic examination revealed a tumour-like mass in the upper quadrants of the right tympanic membrane. Audiometric testing for the right ear showed a type B tympanogram, and pure-tone audiometry indicated mixed hearing loss with an ABG ranging from 20 to 50 dB (Figure 4).

**Figure 5:**
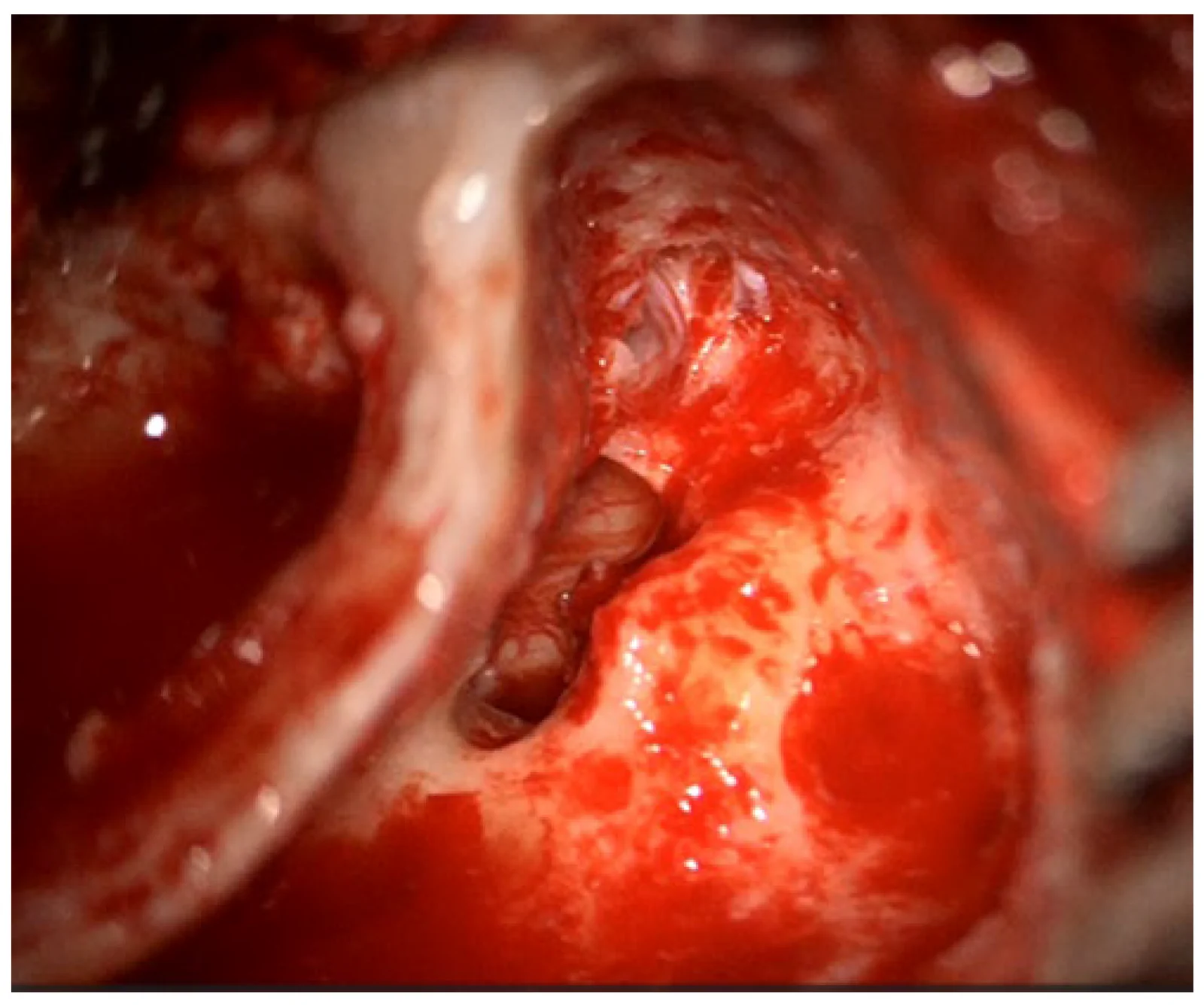
Intraoperative view after tumour removal. Incus and head of the malleus have been removed to facilitate total tumour removal. Incus interposition was used as an ossicular reconstruction.

A canal wall-up mastoidectomy was performed in a mastoid process filled with mucus. A highly vascular reddish tumour was identified in the attic and antrum. A part of the tympanic membrane, along with the tumour filling the tympanic cavity, surrounding the ossicles, and occupying the attic, was removed. Ossiculoplasty with incus interposition and myringoplasty using temporalis muscle fascia were performed (Figure 5).

The Marinelli stage of the tumour was T2c. Histopathological examination confirmed MeNET. Immunohistochemistry was positive for synaptophysin.

### 3.3. Case 3

A 30-year-old female patient (M.K.) was admitted to our department for surgical treatment of a right tympanic cavity mass with a suspected glomus tympanicum (paraganglioma). The initial symptoms had appeared four years earlier, including pulsatile tinnitus in the right ear, primarily after physical exertion and in stressful situations. The patient did not report hearing loss.

**Figure 6:**
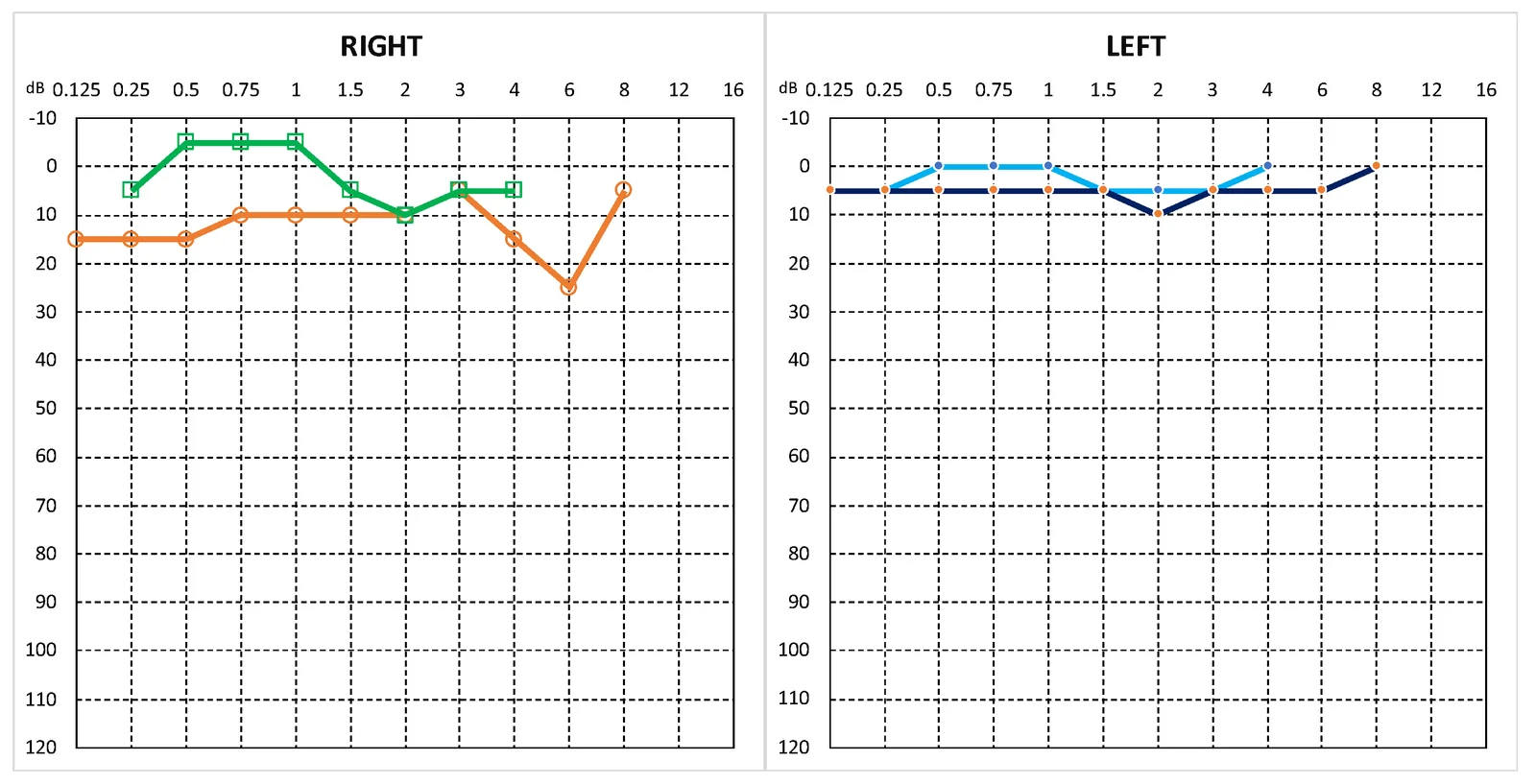
The results of pure-tone audiometry revealed normal hearing thresholds. Green and orange lines represent air-conduction and bone-conduction thresholds of the right ear, respectively. Dark blue and light blue lines represent air-conduction and bone-conduction thresholds of the left ear, respectively.

Pure-tone audiometry showed normal hearing (Figure 6). The patient was scheduled for surgery. A canal wall-up antromastoidectomy and posterior and superior tympanotomy were performed, exposing the right tympanic cavity tumour. The tympanomeatal flap was elevated, revealing a pale, slightly haemorrhagic tumour adherent to and involving the tympanic membrane. The tympanic membrane was carefully dissected free from the lesion. Using a combined surgical approach, the tumour was removed in a piecemeal fashion while preserving the integrity of the ossicular chain.

**Figure 7:**
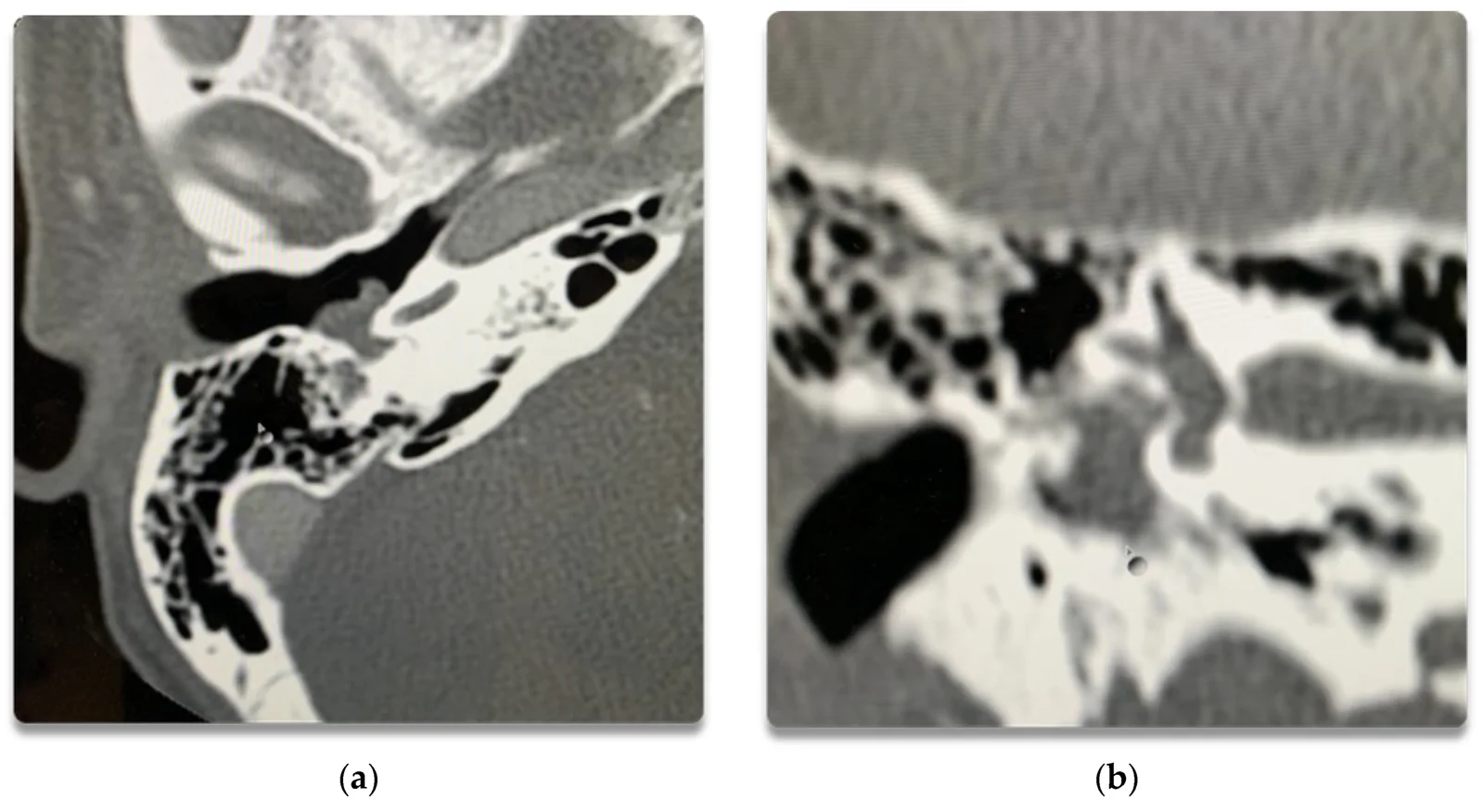
Temporal bone HRCT showing middle ear mass with extension to hypotympanum: (**a**) Axial view; (**b**) Coronal view.

The tumour extended into the sinus tympani. Encasement of the stapes was observed (Figure 7). Based on the intraoperative findings, including tympanic membrane involvement, the lesion was classified as Marinelli stage T2c. Histopathological examination confirmed a grade 1 middle ear neuroendocrine tumour (MeNET G1). Immunohistochemistry demonstrated synaptophysin+, chromogranin+, and the Ki-67 proliferation index was less than 1%.

### 3.4. Case 4

A 40-year-old male patient (J.P.) was admitted to our department following referral from another centre after biopsy of a right middle ear tumour. Histopathological examination revealed a middle ear neuroendocrine tumour. Immunohistochemistry showed synaptophysin+, chromogranin A−, and a Ki-67 proliferation index of approximately 3%. The patient reported right-sided hearing loss persisting for approximately 1.5 years.

**Figure 8:**
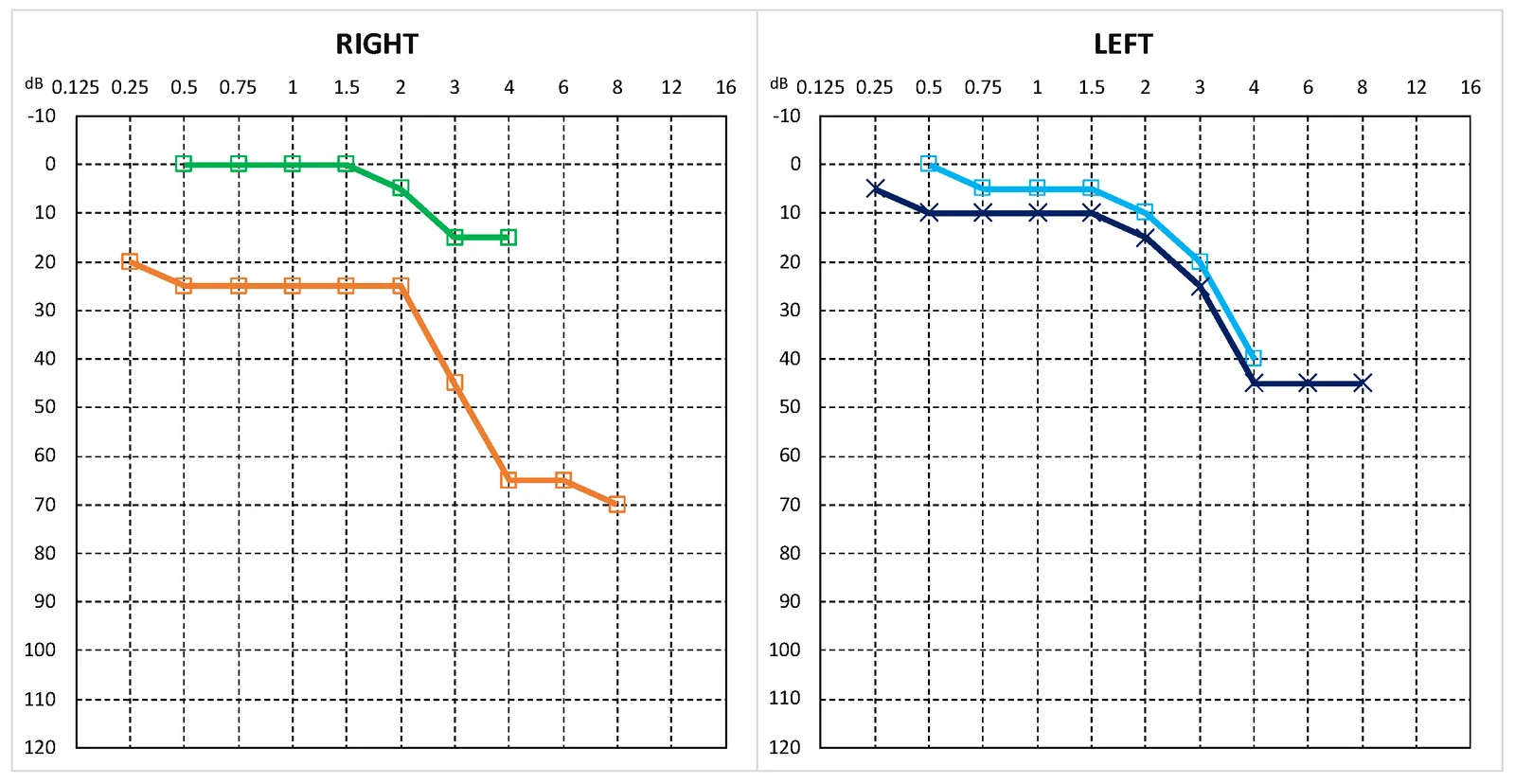
The results of pure-tone audiometry indicated conductive hearing loss in the right ear. Green and orange lines represent air-conduction and bone-conduction thresholds of the right ear, respectively. Dark blue and light blue lines represent air-conduction and bone-conduction thresholds of the left ear, respectively.

**Figure 9:**
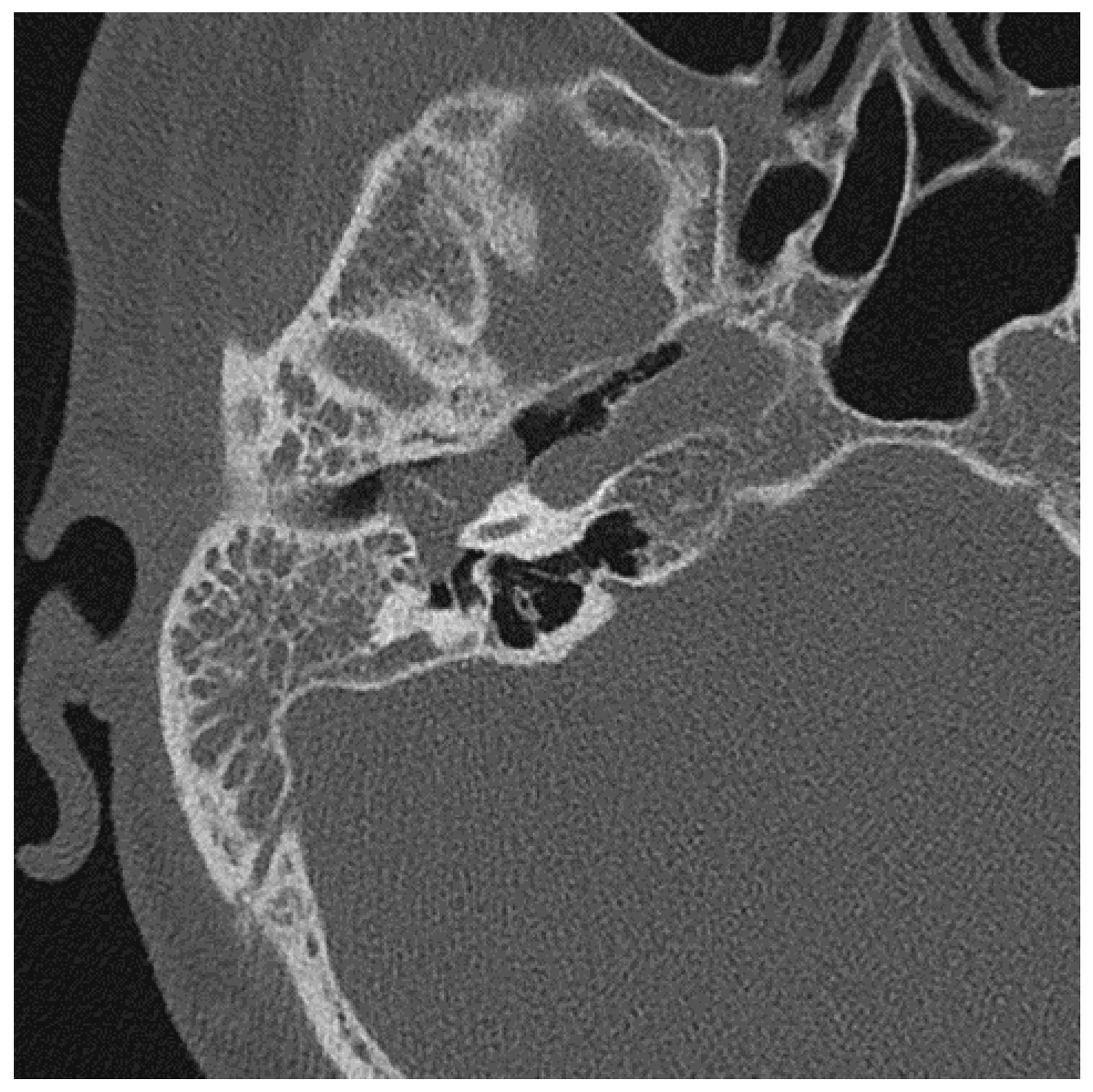
CT scan of the right ear.

Audiometric testing demonstrated conductive hearing loss in the right ear with a 25 dB air–bone gap, consistent with middle ear pathology (Figure 8). High-resolution CT of the temporal bone demonstrated bulging of the tympanic membrane, predominantly in the superior quadrants, complete opacification of the tympanic cavity, and diffuse soft tissue opacification of the mastoid antrum and mastoid air cell system. The ossicular chain remained intact, with no evidence of bony erosion (Figure 9).

**Figure 10:**
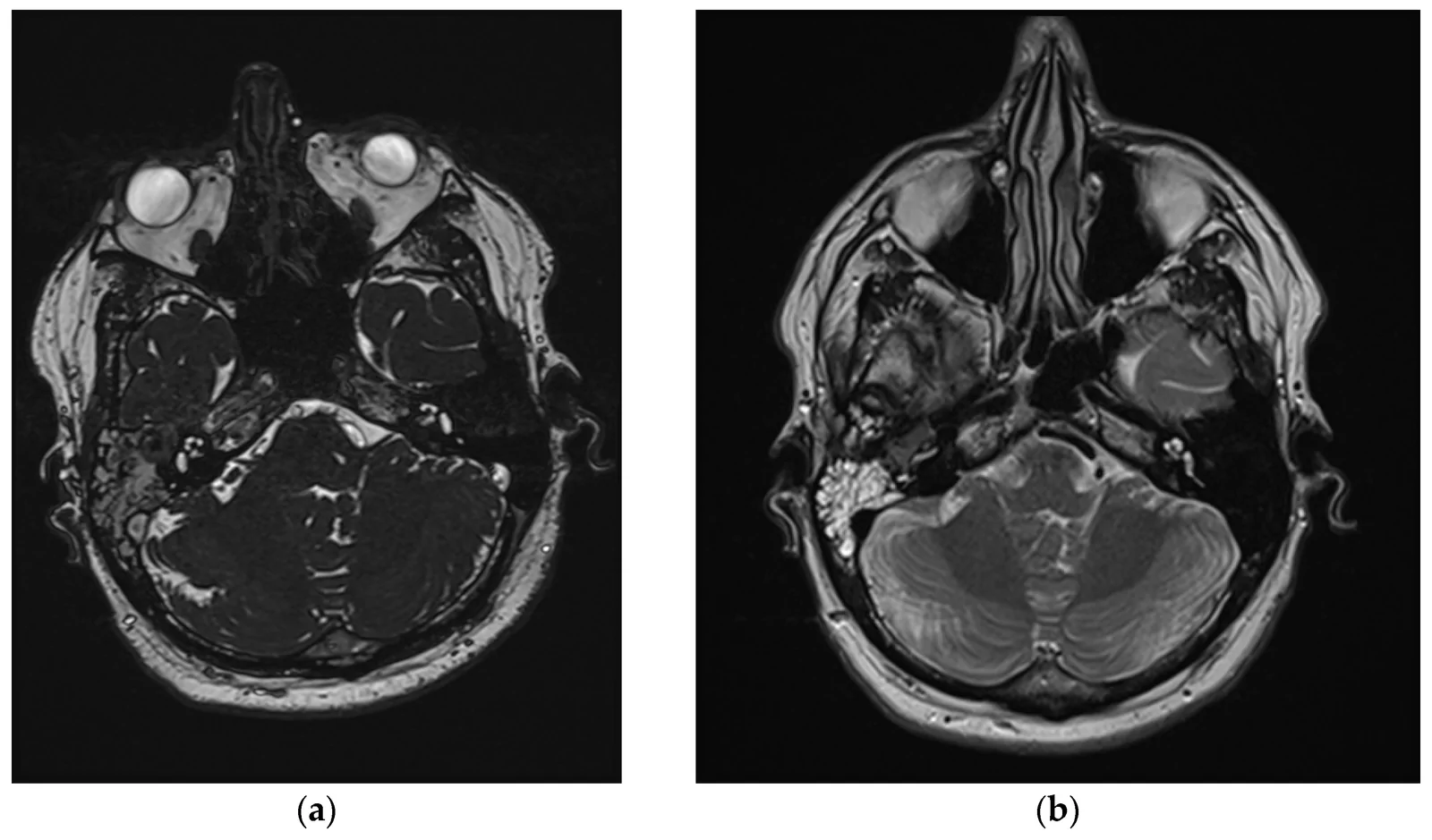
MRI scans of Patient 4 (J.P.): (**a**) T1-weighted sequence; (**b**) T2-weighted sequence.

Contrast-enhanced MRI provided further characterization. The right middle ear cavity was occupied by heterogeneous soft tissue. A distinct soft tissue mass was identified within the effusion, demonstrating hypointense signal on T2-weighted sequences and moderate hyperintensity on T1-weighted images. The lesion encased but did not clearly destroy the ossicular chain (Figure 10).

Metastatic workup, including CT of the chest and abdomen together with 68Ga-DOTA-TATE PET-CT, demonstrated no pathological lymphadenopathy, an absence of distant metastases, and no primary lesions at other neuroendocrine sites. A canal wall-up mastoidectomy was performed in a large mucoid-filled mastoid process. The tumour filled the attic, surrounded the ossicles, and extended into the antrum. Using a combined approach, the friable tumour overlying the tympanic segment of the facial nerve was excised. The tumour occupied the attic and hypotympanum, extended into the supratubal recess, Eustachian tube, and the sinus tympani, and enveloped the stapes. The tumour-involved posterior portion of the tympanic membrane had to be resected and reconstructed.

### 3.5. Case 5

A 19-year-old male patient (P.R.) was admitted to our department with a middle ear neuroendocrine tumour diagnosed in another centre where mastoidectomy and partial removal of the tumour had been performed.

**Figure 11:**
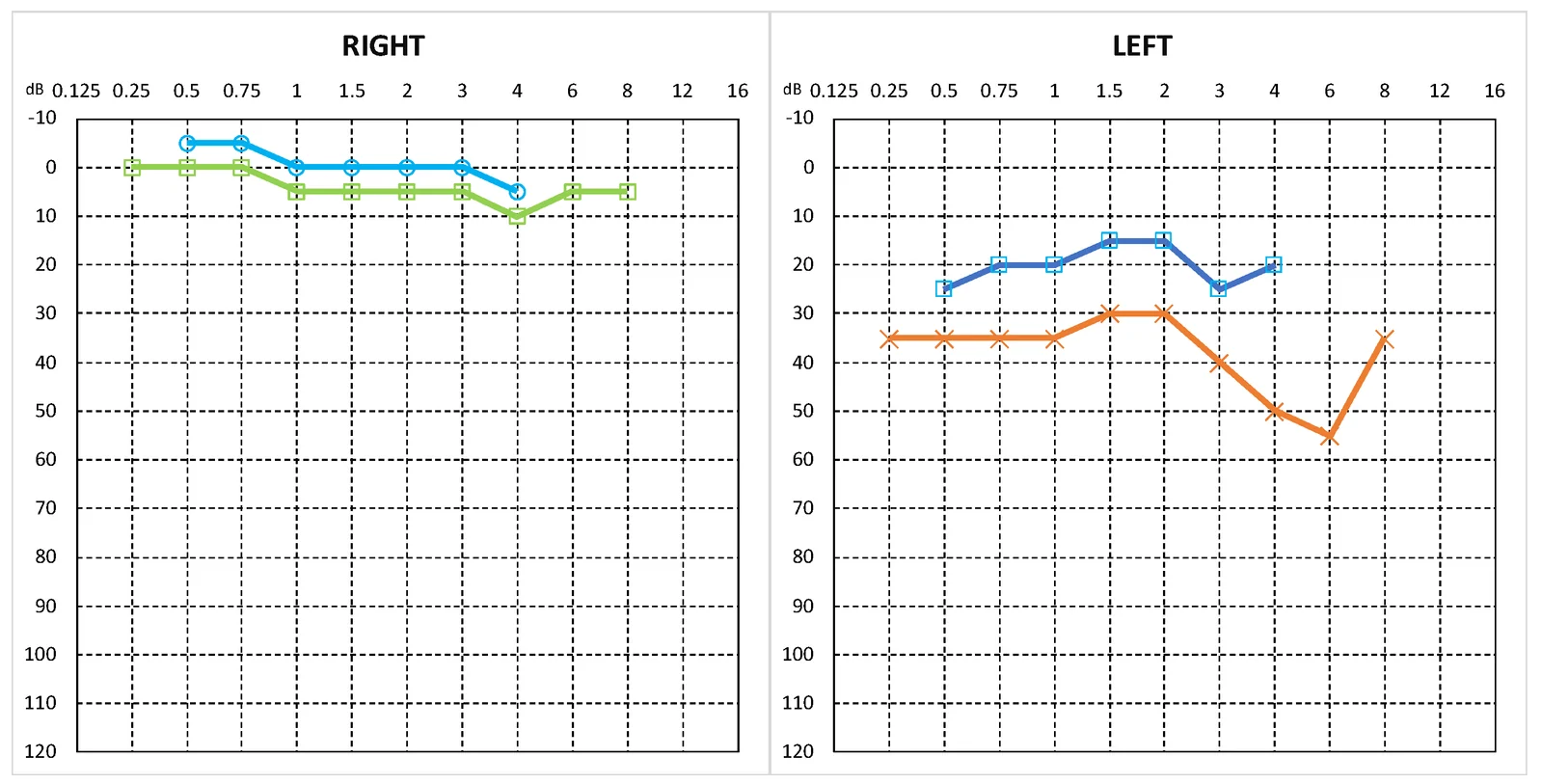
The results of pure-tone audiometry (before second operation) indicated mixed hearing loss in the left ear. Green and orange lines represent air-conduction and bone-conduction thresholds of the right ear, respectively. Dark blue and light blue lines represent air-conduction and bone-conduction thresholds of the left ear, respectively.

**Figure 12:**
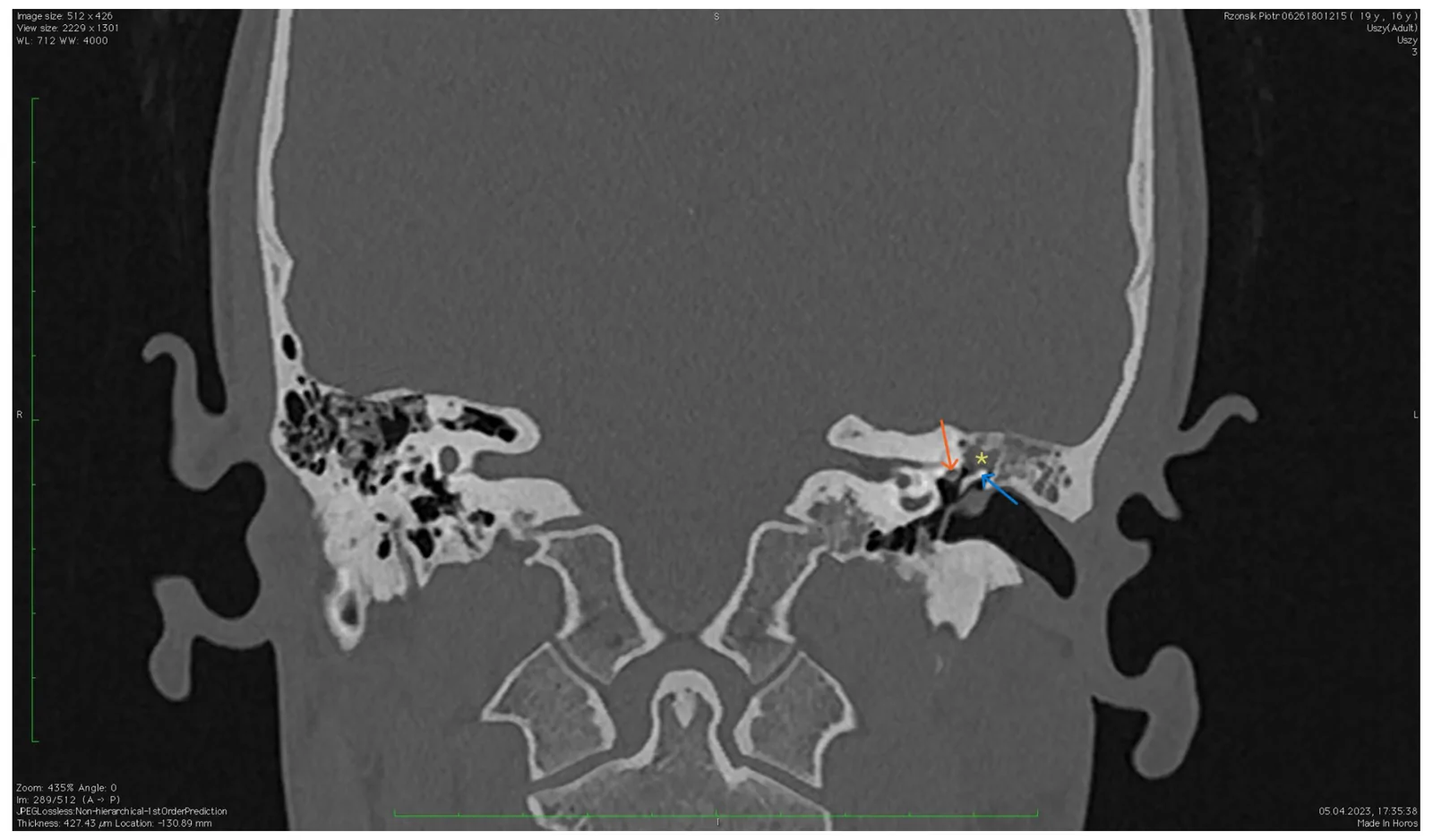
Pre-operative CT (left ear). The image shows a tumour involving the tympanic membrane, attic, and mastoid antrum. *—tumour; blue arrow—head of the malleus; orange arrow—facial nerve.

Preoperative audiometry revealed left-sided conductive hearing loss with a 30 dB air–bone gap and a type B tympanogram (Figure 11). High-resolution CT of the temporal bone demonstrated signs of tympanic membrane retraction and thickening. In the superior part of the tympanic membrane, in continuity with the ossicular chain, a soft tissue mass with irregular contours was visible. Similar soft tissue densities were noted in the tegmen tympani. No features of ossicular chain destruction were identified (Figure 12).

MRI demonstrated the following findings: In the left middle ear, within the epitympanum and Prussak’s space, a structure exhibiting contrast enhancement was visible, contiguous with a soft tissue lesion involving the mastoid antrum and mastoid air cell system. The lesions were isointense on T1- and T2-weighted images. Otoscopic examination revealed the external auditory canal was completely filled with a polypoid lesion. The mass was densely adherent to both the tympanic membrane and the canal skin. A canal wall-up mastoidectomy with wide posterior and superior tympanotomy was performed. Histopathological examination confirmed MeNET G1. Immunohistochemistry demonstrated: synaptophysin+, chromogranin A equivocal, CK+.

## 4. Discussion

Historically, middle ear neuroendocrine tumours have been described under a variety of terms, including middle ear adenoma, carcinoid tumour of the middle ear, adenomatous tumour of the middle ear, and neuroendocrine adenoma of the middle ear [11,12]. This diversity of nomenclature reflects the longstanding debate regarding their histogenesis and biological behaviour. Contemporary concepts increasingly support a unified neuroendocrine entity encompassing the entire spectrum of these lesions. Accordingly, in the present study, we consistently use the term Middle Ear Neuroendocrine Tumour (MeNET), in line with recent literature and current concepts regarding these neoplasms [1,2].

Middle ear neuroendocrine tumours are rare and complex neoplasms that require a multidisciplinary diagnostic and therapeutic approach [13,14]. It is important to emphasize that the symptoms of MeNET, such as hearing loss, tinnitus, or an aural fullness, are nonspecific and may mimic other, more common middle ear pathologies, significantly complicating early diagnosis [15]. In our series, the clinical presentation was dominated by conductive hearing loss, observed in 80% of patients (4/5), while pulsatile tinnitus (1/5), otorrhea (1/5), and aural fullness (1/5) were less frequent. Therefore, it is crucial to include MeNET in the differential diagnosis of patients with chronic ear symptoms, particularly when standard treatment does not yield the expected results.

Marinelli et al. developed a TNM classification system for MeNETs [16]. T1 refers to a tumour confined to the middle ear cavity that does not surround or firmly adhere to the ossicles. T2a denotes a tumour filling the middle ear cavity, which surrounds the ossicles with or without erosion. T2b describes a tumour involving the mastoid process. A T2c tumour additionally infiltrates the tympanic membrane and extends into the external auditory canal. T3 describes a tumour that adheres to critical neurovascular structures, such as the petrous segment of the internal carotid artery, the facial nerve, the sigmoid sinus, or the dura mater. A tumour that invades the intradural space is classified as T4. In our study, tumour staging was classified as T2b in one case (patient A.P.), T2c in three cases (patient P.M., M.K., P.R.), and T3 in one case (patient J.P.) (Table 1). The assigned stages were based on the extent of tumour involvement documented on preoperative imaging and confirmed during surgical exploration. The rationale for staging in individual patients is summarized in Table 1.

Historically, Saliba and Evrard proposed a three-tier classification of middle ear glandular tumours based on immunohistochemical findings and metastatic potential [17]. However, current concepts increasingly support a unified neuroendocrine entity encompassing the entire spectrum of these lesions. Accordingly, the WHO Classification of Head and Neck Tumours (5th edition, 2022) recognizes these neoplasms within the spectrum of middle ear neuroendocrine tumours (MeNETs), emphasizing their shared epithelial and neuroendocrine differentiation [1]. More recently, the MANET concept (Mixed Adenoma and Well-Differentiated Neuroendocrine Tumour of the Middle Ear) has further supported the view that these lesions represent a single clinicopathological entity with variable histological features rather than distinct tumour categories [2].

Histopathologically, MeNET demonstrates considerable heterogeneity. Tumours may exhibit glandular, trabecular, solid, or mixed growth patterns. The coexistence of epithelial and neuroendocrine differentiation represents the hallmark feature of these lesions [14,18]. Immunohistochemistry, therefore, plays a crucial role not only in confirming the diagnosis but also in distinguishing MeNET from other middle ear neoplasms.

In our series, synaptophysin expression was observed in all five cases (100%), confirming neuroendocrine differentiation in all tumours. Chromogranin A was positive in two patients, negative in one patient, equivocal in one patient, and not assessed in one patient. CD56 expression was evaluated in one patient and showed positive staining. The Ki-67 proliferation index was available in four cases and ranged from less than 1% to 3%, indicating low proliferative activity. Overall, these immunohistochemical findings are consistent with the generally indolent biological behaviour and low-grade nature of middle ear neuroendocrine tumours (MeNETs) [2].

The differential diagnosis includes paraganglioma, middle ear adenoma without neuroendocrine differentiation, adenocarcinoma, and metastatic neuroendocrine neoplasms. In contrast to paraganglioma, MeNET demonstrates co-expression of epithelial markers such as cytokeratins together with neuroendocrine markers, including synaptophysin and chromogranin A. Histopathological evaluation, combined with immunohistochemistry, therefore, remains essential for accurate diagnosis.

One of the major diagnostic challenges associated with MeNET is the considerable overlap between radiological findings and other middle ear pathologies. In daily clinical practice, these lesions are frequently misdiagnosed preoperatively as paraganglioma, cholesteatoma, chronic otomastoiditis, or middle ear adenoma without neuroendocrine differentiation. In our series, the initial diagnosis included suspected paraganglioma in two patients, highlighting the nonspecific nature of both clinical and imaging findings.

Computed tomography remains the preferred initial imaging modality because it allows precise evaluation of middle ear anatomy and tumour extension [19,20]. In most cases, MeNET appears as a soft tissue mass occupying the tympanic cavity with variable extension into the mastoid air cells [10,17,19]. Importantly, unlike cholesteatoma, extensive bone destruction is uncommon despite the relatively large tumour size [10,21,22]. Preservation of ossicular integrity and the absence of erosive changes, therefore, may suggest MeNET in selected cases. Nevertheless, ossicular encasement is frequently observed and may contribute to conductive hearing loss.

MRI contributes substantially to differential diagnosis because of its superior soft tissue resolution [19,20]. MeNETs usually demonstrate contrast enhancement and relatively low T2 signal intensity compared with inflammatory lesions. However, differentiation from glomus tympanicum remains difficult because both entities may exhibit vivid enhancement. Hypervascularity and “salt-and-pepper” appearance, characteristic for paragangliomas, are usually absent in MeNETs. Diffusion-weighted imaging may additionally assist in excluding cholesteatoma, which typically demonstrates diffusion restriction [19,20]. The principal radiological findings observed in our cohort are summarized in Table 2.

Although MeNET is generally considered an indolent tumour with predominantly localized behaviour, rare cases of locally aggressive disease and distant metastases have been reported in the literature, particularly in tumours corresponding to type III according to the historical Saliba classification. Fundakowski et al. [23] described the first reported case of distant osseous metastasis involving the cervical lymph nodes and iliac crest. Metastatic spread to regional lymph nodes, lungs, and bones has subsequently been documented in rare instances [24]. The metastatic potential appears to correlate with adverse histopathological features, including increased mitotic activity and a Ki-67 proliferation index greater than 5% [21]. Although the overall risk remains low, these findings support complete surgical excision with clear margins and long-term postoperative surveillance [25].

The role of nuclear medicine imaging in MeNET remains incompletely established [21]. Somatostatin receptor imaging, including 68Ga-DOTA-TATE PET-CT, may be useful in selected patients with suspected recurrence, metastatic disease, or high proliferative index. As part of postoperative staging, all patients underwent 68Ga-DOTA-TATE PET-CT, which did not demonstrate regional lymph node involvement or distant metastatic disease. However, the role of somatostatin receptor imaging in the routine surveillance of MeNET remains to be fully established, although it may improve long-term follow-up in selected patients with more aggressive histopathological features [26].

The treatment of choice for MeNET is surgical resection of the tumour, with the choice of surgical technique depending on the stage of the tumour, its relationship to surrounding anatomical structures, and the possibility of preserving hearing. The surgical approach was selected individually for each patient based on preoperative imaging findings, particularly tumour location and extent, with the primary goal of achieving complete tumour excision while preserving critical anatomical structures whenever feasible. The completeness of tumour removal was assessed intraoperatively by the operating surgeon based on macroscopic evaluation of the surgical field. Whenever oncologically safe, preservation of the ossicular chain and hearing function was attempted; however, oncological radicality remained the primary surgical objective. In our study, we highlighted the importance of radical tumour excision to minimize the risk of recurrence [17]. In our series, canal wall-up mastoidectomy combined with posterior and superior tympanotomy allowed adequate visualization and radical removal in the majority of patients. Ossicular preservation was feasible in selected cases; however, tumour encasement occasionally required partial ossicular chain removal and reconstruction.

The literature indicates that recurrence most commonly results from incomplete excision rather than intrinsic tumour aggressiveness [16,17]. Consequently, meticulous intraoperative assessment of hidden recesses, including the sinus tympani, hypotympanum, supratubal recess, and Eustachian tube orifice, is essential [17,21,27]. These regions may harbour microscopic residual disease that later contributes to recurrence.

However, due to the propensity of MeNET for recurrence and the potential risk of metastasis, long-term follow-up of patients is essential. In the context of post-treatment monitoring, regular otoscopic, audiological, and imaging examinations are recommended. Imaging studies such as computed tomography (CT) and magnetic resonance imaging (MRI) play a key role in assessing potential recurrences or metastases. Additionally, in patients at high risk of disease progression, functional imaging studies such as somatostatin receptor scintigraphy may be justified [9,28]. None of our patients exhibited evidence of local recurrence nor regional or distant metastases during the follow-up period.

## 5. Conclusions

Middle ear neuroendocrine tumours are rare neoplasms characterized by nonspecific clinical presentation and considerable diagnostic challenges. The treatment of choice is surgical intervention using an appropriate surgical technique, with preservation of hearing whenever feasible. Although no recurrence or metastases were observed in our cohort, the relatively short follow-up in some patients and previously reported late recurrences in the literature justify long-term surveillance. Increased awareness of MeNET among otolaryngologists, radiologists, and pathologists may contribute to earlier diagnosis and improved long-term outcomes.

## Figures and Tables

**Table 1:** Clinical, pathological, treatment, and follow-up characteristics of patients with middle ear neuroendocrine tumours (MeNETs).

Patient	Sex	Age	Symptoms	Treatment	Marinelli Stage	Basis for Staging	Pathology Report	IHC	Follow-Up	Postoperative Hearing
A.P.	female	38	aural fulness, conductive hearing loss	CWU mastoidectomy	T2b	Mastoid extension	MeNET G1	ChrA+, Syn+, CD56+, Ki-67 ≈ 2%	3 y	Normal hearing
									PET-CT: negative	
P.M.	male	49	mixed hearing loss	CWU mastoidectomy + ossiculoplasty (incus interposition) + myringoplasty	T2c	Tympanic membrane involvement	MeNET	Syn+	6.5 y	Mixed HL (ABG 20 dB)
									PET-CT: negative	
M.K.	female	30	pulsatile tinnitus, normal hearing	CWU mastoidectomy + posterior and superior tympanotomy	T2c	Tympanic membrane involvement	MeNET G1	Syn+, ChrA+, Ki-67 < 1%	4 y	Normal hearing
									PET-CT: negative	
J.P.	male	40	conductive hearing loss	CWU mastoidectomy + posterior and superior tympanotomy	T3	Facial nerve involvement	MeNET	Syn+, ChrA−, Ki-67 ≈ 3%	1 y	Conductive HL (ABG 15–20 dB)
									PET-CT: negative	
P.R.	male	19	otorrhea and conductive hearing loss	CWU mastoidectomy + posterior and superior tympanotomy	T2c	Tympanic membrane and EAC involvement	MeNET G1	Syn+, ChrA+/−, CK+	8 months	Mixed HL (ABG 10–15 dB)
									PET-CT: negative	

Abbreviations: ABG, air–bone gap; ChrA, chromogranin A; CK, cytokeratin; CWU, canal wall-up; EAC, external auditory canal; HL, hearing loss; IHC, immunohistochemistry; MeNET, middle ear neuroendocrine tumour; PET-CT, positron emission tomography–computed tomography; Syn, synaptophysin.

**Table 2:** Summary of radiological findings in patients with middle ear neuroendocrine tumour (MeNET).

Patient	HRCT Findings	MRI Findings
A.P.	Middle ear soft tissue mass with mastoid extension; no ossicular destruction or bony erosion	T1 hyperintense, T2 hypointense lesion with heterogeneous contrast enhancement
P.M.	Soft tissue mass involving the attic and tympanic cavity; no bony erosion	Not performed
M.K.	Soft tissue mass extending into the sinus tympani with stapes encasement	Not performed
J.P.	Complete soft tissue opacification of the middle ear and mastoid air cells; intact ossicular chain; no bony erosion	T2 hypointense, T1 moderately hyperintense enhancing lesion encasing the ossicles
P.R.	Soft tissue mass involving the tympanic membrane, attic, mastoid antrum and external auditory canal; no ossicular destruction	Contrast-enhancing lesion involving the epitympanum and mastoid air cells

## Data Availability

The data presented in this study are available from the corresponding author upon reasonable request. The data are not publicly available due to privacy and ethical restrictions.

## Abbreviations

The following abbreviations are used in this manuscript:

MeNET	Middle ear neuroendocrine tumour
CT	Computed tomography
MRI	Magnetic Resonance Imaging
SI	Signal intensity
T1W	T1-weighted sequences
T2W	T2-weighted sequences
ABG	Air–bone gap
